# A spatio-temporal continuous soil moisture dataset over the Tibet Plateau from 2002 to 2015

**DOI:** 10.1038/s41597-019-0228-x

**Published:** 2019-10-31

**Authors:** Yaokui Cui, Chao Zeng, Jie Zhou, Hongjie Xie, Wei Wan, Ling Hu, Wentao Xiong, Xi Chen, Wenjie Fan, Yang Hong

**Affiliations:** 1 Institute of Remote Sensing and GIS, School of Earth and Space Sciences, Peking University, Beijing, 100871 China; 2 Beijing Key Laboratory of Spatial Information Integration & Its Applications, Beijing, 100871 China; 3 School of Resource and Environment Science, Wuhan University, Wuhan, 430072 China; 4 State Key Laboratory of Remote Sensing Science, Institute of Remote Sensing and Digital Earth, Chinese Academy of Sciences, Beijing, 100101 China; 5 Department of Geological Sciences, University of Texas at San Antonio, San Antonio, TX 78249 USA; 6 Department of Civil Engineering and Environmental Science, University of Oklahoma, Norman, 73019 OK United States

**Keywords:** Hydrology, Environmental impact, Climate sciences

## Abstract

Surface soil moisture is a key variable in the exchange of water and energy between the land surface and the atmosphere, and critical to meteorology, hydrology, and ecology. The Tibetan Plateau (TP), known as “The third pole of the world” and “Asia’s water towers”, exerts huge influences on and sensitive to global climates. In this situation, longer time series of soil moisture can provide sufficient information to understand the role of the TP. This paper presents the first comprehensive dataset (2002–2015) of spatio-temporal continuous soil moisture at 0.25° resolution, based on satellite-based optical (i.e. MODIS) and microwave (ECV) products using a machine learning method named general regression neural network (GRNN). The dataset itself reveals significant information on the soil moisture and its changes over the TP, and can aid to understand the potential driven mechanisms for climate change over the TP.

## Abstract (summary)

**Table:** 

Measurement(s)	wetness of soil
Technology Type(s)	computational modeling technique
Factor Type(s)	land surface temperature • normalized difference vegetation index • albedo • digital elevation model
Sample Characteristic - Environment	soil
Sample Characteristic - Location	Tibetan Plateau

Machine-accessible metadata file describing the reported data: 10.6084/m9.figshare.9925067

## Background & Summary

Land surface soil moisture is a key variable in the exchange of water and energy between the land surface and the atmosphere, and critical to meteorology, hydrology, and ecology^1,2^. Soil moisture can impact runoff and landslide generation, drought development and be used in many other hydrological, meteorological and ecological applications^1–3^. A longer time series of spatially consistent and temporally continuous soil moisture products can improve our understanding of meteorological and hydrological processes and associated modelling at the daily timescale, and it is also very useful for a number of applications such as weather forecasting and drought monitoring^4^.

A longer time series of spatially consistent and temporally continuous soil moisture products can be utilized for more accurate and reliable estimates of deeper soil moisture, and evapotranspiration etc., also be useful for a number of applications such as data assimilation, weather forecasting and drought monitoring^4^, and eventual improve our understanding of feedback mechanisms between different meteorological and hydrological components, especially under the background of climate change.

**Fig. 1:**
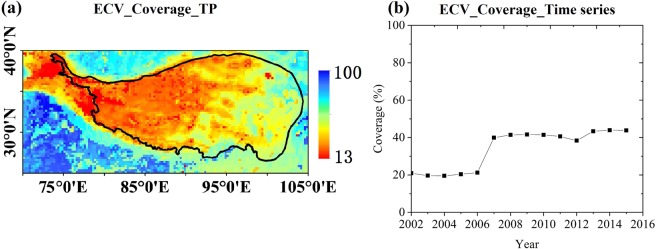
Coverage of the original ECV soil moisture product. (**a**) The spatial distribution over 2002–2015. (**b**) The time series from 2002 to 2015.

The Tibetan Plateau (TP), known as the Earth’s Third Pole and Asia’s water towers, plays an important role in global change. Rivers originated in the TP support the development of society and economy in the surrounding countries. However, the Tibetan Plateau is sensitive to climate change and human activities, making it necessary to obtain a comprehensive and long-term observation covering all water cycle components (including soil moisture) over this region. Due to the water body, glacier, frozen soil and vegetation, the soil moisture retrieval algorithm is not always suitable^5^. For the uncertainty of forcing data, such as precipitation etc., the process-based model is difficult to simulate the surface soil moisture. Until to now, there are no spatio-temporal continuous soil moisture product to meet the need of application of meteorology, hydrology and ecology, also limiting the continuous analysis of its spatiotemporal variation^6^. There are several remote sensing based soil moisture products^7,8^, such as FY-3, ASCAT, SMOS, AMSR-E, AMSR2, and SMAP with spatial resolution of larger than tens of kilometres. According to the previous studies, the percentage of these data gaps over the TP is more than 40%, and even more than 80% in the central and western TP^9^. Hence, the low coverage of these products over the TP located in low altitude region is the major problem for researches and applications. To overcome this disadvantage of remote sensing based soil moisture product, ECV combined soil moisture product (version ESA CCI SM v04.2, hereafter called ECV product) uses almost all of the available satellites including active and passive observations to produce longer time series of soil moisture. The ECV product is the first purely multi-decadal satellite-based soil moisture product that spans 38 years (from 1978 to 2016) on a daily basis and at a spatial resolution of 0.25°. ECV is also the first long time series of remote sensing based soil moisture product and has very good consistency. ECV product was developed as part of the European Space Agency’s (ESA) Water Cycle Multi-mission Observation Strategy (WACMOS) and Soil Moisture Climate Change Initiative (CCI) projects^10,11^. However, it is unfortunate that the coverage is not significantly improved as expected, especially over the TP (Fig. 1(a,b)). The satellite orbit, vegetation, frozen soil, snow cover and glaciers are the main factors that making the soil moisture retrieval algorithm does not work well. Hence, the released original ECV product only represents the soil moisture under suitable condition. Until to now, there is no spatio-temporal continuous and open-access remote sensing based soil moisture product across the TP.

**Fig. 2:**
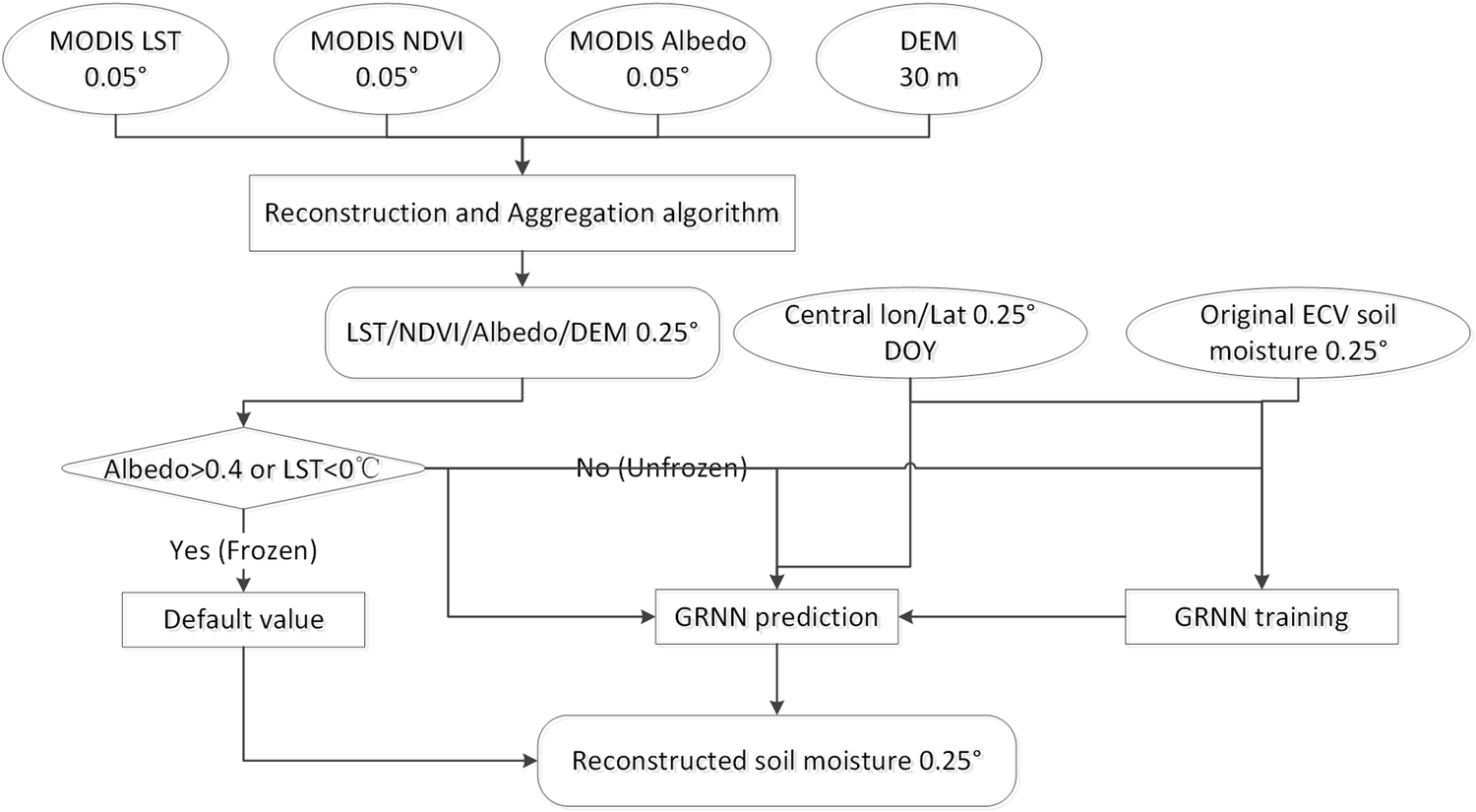
Flowchart for producing spatio-temporal continuous soil moisture dataset based on General Regression Neural Network (GRNN) method using ECV product.

In this study, we use MODIS (Moderate Resolution Imaging Spectroradiometer) Land Surface Temperature (LST) daily product (MOD11C1, 0.05°), Normalized Difference Vegetation Index (NDVI) product (MOD13C1, 0.05°) and albedo product (MOD43C1, 0.05°), the DEM (30 m) provided by NASA Shuttle Radar Topographic Mission (SRTM), and the ECV V04.2 combined soil moisture product (Daily, 0.25°), as the main data sources. The algorithm is a modified version of the published method^9^, using the General Regression Neural Network (GRNN). The flowchart for producing and validating this dataset is shown in Fig. 2. This newly generated dataset could be valuable in addressing scientific questions associated with global change, land-atmosphere interaction, ecological evolution, etc.

## Methods

The method used in this paper is a modified version of the method proposed by Cui^9^, with the BP-NN being replaced by the GRNN model, since there are too many gaps for the ECV product over 2002–2006 and GRNN could better deal with this situation having limited training data. In addition, the GRNN has less parameters and better generalization ability. A stepwise processing method is used to obtain the final soil moisture products (Fig. 2) and is described in detail as follows.

### Data Pre-processing

MODIS LST, NDVI and albedo with 0.05°resolution are reconstructed using multi-temporal robust regression method^12^, i.e., the HANTS method (Harmonic Analysis of Time Series)^13^, and the statistical method based on temporal filtering, respectively. More detailed information also could be found in the reference of Cui^9^. These three reconstructed methods could not only fill the gap in the original data, but also improve the quality. Afterwards, the LST, NDVI, albedo and DEM are resampled from 0.05° or 30 m to 0.25°, to be used as inputs together with the latitude, longitude, and DOY (day of year).

### Soil moisture reconstructing

**Fig. 3:**
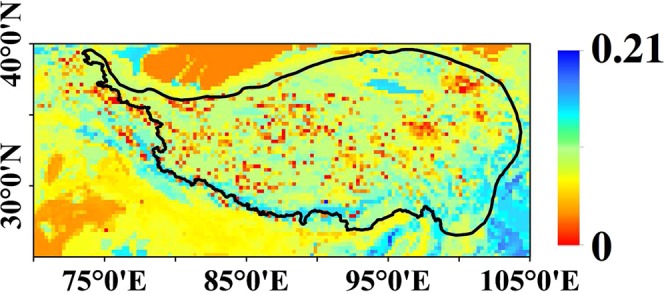
Soil moisture default value used in this study when the soil is frozen, unit: cm^3^ cm^−3^.

When the soil is unfrozen, the GRNN method is used to finish the reconstructing process. When the soil is frozen, the default value (Fig. 3) is used to fill the gaps. For the unfrozen soil, the process has two steps: training and predicting.

#### Frozen-unfrozen soil condition

For only the liquid water in the soil could be observed by remote sensing sensor, distinguishing different soil condition is necessary for the reconstruction process, where a default value is used under frozen soil condition and GRNN-based method is used under unfrozen soil condition. The frozen soil condition defined as the land covered by snow or the LST lower than 0 °C, where the soil moisture is nearby the residual water content. For simplicity, we use MODIS albedo (threshold is 0.4) and LST (threshold is 0 °C) product to distinguish whether the soil is frozen or not. In this paper, the unfrozen soil condition means that the LST is higher than 0 °C and there is no snow covered (albedo smaller than 0.4), where there are strong relationship between soil moisture and remote sensed LST, NDVI, albedo, etc.

#### GRNN model training

The pixels that have available value for both optical and ECV products are extracted to form a training dataset, 2° by 2° and year by year. A GRNN with spread of radial basis functions of 0.05 is built in Matlab R2016b version. As all parameters, including LST, NDVI, albedo, DEM, latitude, longitude, and DOY, are selected when the ECV product has value, and then the GRNN was trained. At last, a trained GRNN was obtained.

#### GRNN model predicting

The complete time series of the seven input variables together with the trained GRNN model are used to generate complete time series of soil moisture.

#### Correction

The mean bias between the original and reconstructed soil moisture is calculated pixel by pixel where the soil under unfrozen soil condition, and then be added to the reconstructed soil moisture to correct the offset.

### Post-processing

When the soil in frozen condition or covered by glacier, the GRNN method is not applicable. Instead, we use a default value to finish the reconstructing process, since the soil moisture has little variation around a default value (residual soil moisture, Fig. 3). To overcome the lack of effective observations, the default value was taken as the smaller one in the minimum value of time series data^9^ and the volumetric water content at −1500 KPa in the soil map^14^. To mitigate the outlier effect, a mean filter every 3 days is applied at pixel scale, when the LST is lower than 0 °C.

## Data Records

The dataset includes not only the reconstructed soil moisture, but also the original ECV soil moisture and auxiliary data, such as: reconstructed LST, reconstructed NDVI, reconstructed albedo, and DEM etc. (shown in Table 1). The data set can be accessed at 10.6084/m9.figshare.7996448 ^15^ (the file is in ‘.rar’ format, compression software is needed to decompress). Files are stored in GeoTiff format and are projected in World Geodetic System 1984 (WGS84). All variables are located in latitude 25°–40° N and longitude 70°–105° E, with spatial resolution of 0.25°. Original ECV soil moisture data is stored in the subfolder named ‘Raw’. An example file name is ‘YYYY_DOY_ECV_Raw.tif’, with YYYY, DOY and Raw standing for year, day of year and original, respectively. The reconstructed soil moisture and quality control data are stored in the subfolder named ‘filled’. An example file name is ‘YYYY_DOY_ECV_filled.tif’ and ‘YYYY_DOY_QC.tif’, with ‘filled’ and ‘QC’ standing for reconstructed and quality control, respectively. The reconstructed LST, NDVI, albedo are stored in the subfolder named ‘Auxiliary’, the file name is similar only with suffix of LST, NDVI, and albedo respectively. Auxiliary data are also included in the subfolders of ‘Dem’ and ‘DefalutV’ (default soil moisture value for soil in frozen condition), the name is just the same as the folder’s name, for there is only one file in the folder. It is noted that each variable appears stored in a subfolder named year by year.

**Table 1:** Data organizations and descriptions for the generated dataset.

Folder	Subfolder	File name	Description
Raw	SM_Ori	YYYY_DOY_ECV_Raw.tif	• Original Soil moisture • Daily • Unit: cm^3^ cm^−3^
Filled	SM_Rec	YYYY_DOY_ECV_Filled.tif	• Reconstructed Soil Moisture • Daily • Unit: cm^3^ cm^−3^
	QC	YYYY_DOY_QC.tif	• Quality control data • 0: Reconstruction using GRNN during soil unfrozen condition • 1: Gaps filled using default value during soil frozen condition • 2: Filtered results
Auxiliary	LST	YYYY_DOY_lst_Filled.tif	• LST: Reconstructed • Unit: K
	NDVI	YYYY_DOY_ndvi_Filled.tif	• NDVI: Reconstructed • Range: 0–10000
	Albedo	YYYY_DOY_albedo_Filled.tif	• Albedo: Reconstructed • Range: 0–10000
	Dem	Dem.tif	• DEM • Unit: °
	DefaultV	DefaultV_TP.tif	• Soil moisture default value • Unit: cm^3^ cm^−3^

## Technical Validation

### Quality control of the production method

The production method is carried out based on the recently published algorithm^9^. The reconstruction methods for optical products as inputs are carried out using the published algorithms of the co-authors (Chao Zeng, Jie Zhou), and each algorithm strictly follows their original workflow. Therefore, the main error sources include errors in the input data i.e., the MODIS and ECV datasets, and on the assumption of soil freezing when the LST is lower than 0 °C. The first error source is primarily due to the retrieval process including atmospheric correction and empirical parameters in retrieval algorithm. The using of several variables as inputs into GRNN could minimize the error effect from single variable. Meanwhile, the aim of this paper is to reproduce a consistent dataset with the original ECV product, only to improve the temporal and spatial continuity. The effect of the second error is mitigated using filter method in the post process.

### Comparison with the original ECV products

**Fig. 4:**
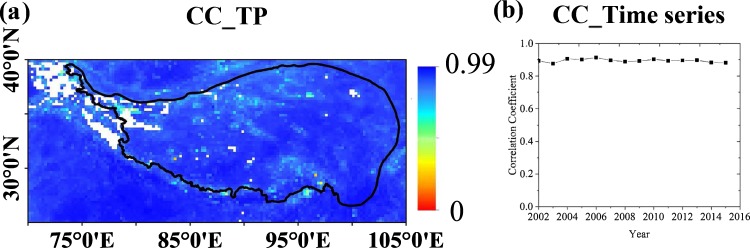
Correlation coefficient (CC) between the reconstructed and original ECV products. (**a**) Spatial distribution over 2002–2015. (**b**) Time series from 2002 to 2015.

The correlation coefficient (CC) between the reconstructed and original ECV data is calculated and shown in Fig. 4(a). In more than 99% available area, the CC is greater than 0.7. The yearly area-averaged CC from 2002 to 2015 shows enough stability (Fig. 4(b)). We can see that the reconstructed data is consistent with the original data, meaning that the trained GRNN model has enough sufficient representativeness and robustness. Hence, the reconstructed product is from the outputs of GRNN model instead of the original one. Which makes the dataset not only having better consistency, but also with the comparable accuracy as the original ECV data. However, it should be noted that the filled values only represent liquid water in the soil, especially soil in frozen condition.

### Comparison with *in situ* measurements

For verification purposes, two nested Soil Moisture/Temperature Monitoring Networks on the central TP (TP-SMTMN)^9,16^, located in the Naqu with 4500 m above the sea level is used. The larger network is 1° × 1° (4 × 4 pixels, 91.5–92.5°E, 31–32°N) containing 38 soil moisture measurement stations and the smaller one is 0.25° × 0.25° (one pixel, 91.75–92°E, 31.5–31.75°N) containing 9 stations. For each station, the soil moisture of the 0–5 cm topsoil was measured with the interval of 30 minutes, and the daily average value is used to validate the original and reconstructed ECV satellite soil moisture.

**Fig. 5:**
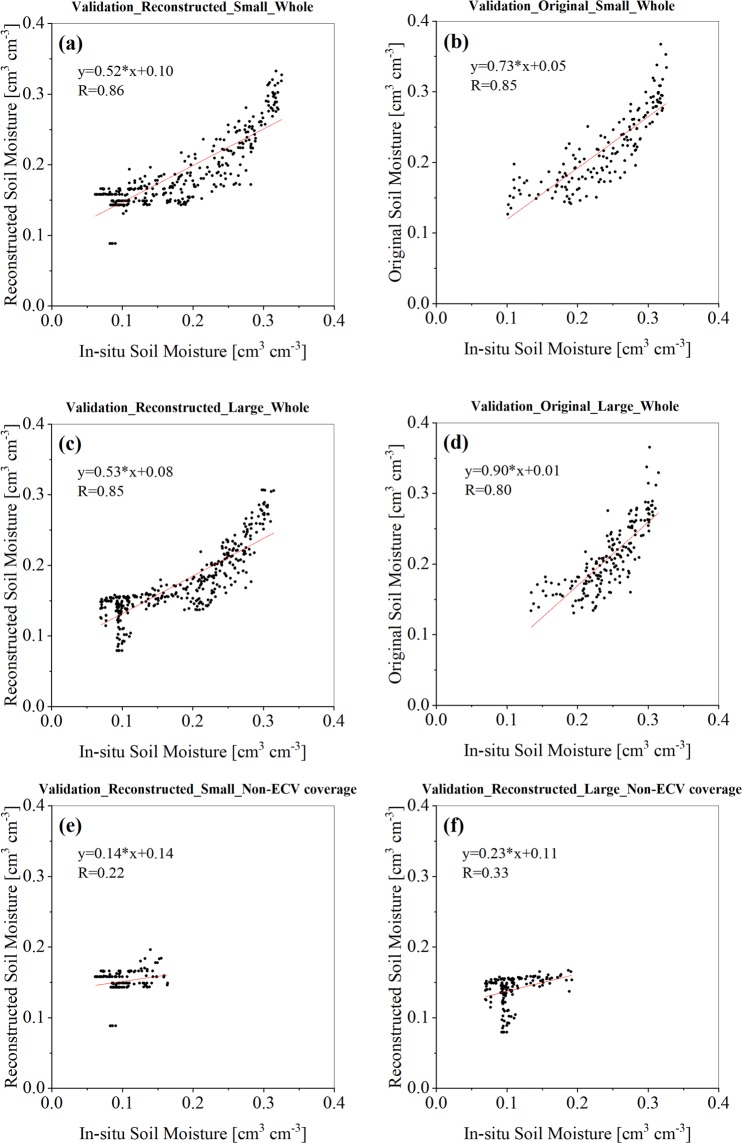
Validation using the field measurements. (**a**,**b**) is results of reconstructed and original products at the small grid, respectively and (**c**,**d**) is results of reconstructed and original products at the large grid, respectively. (**e**,**f**) is results of reconstructed products at Non-ECV coverage period for small and large grid, respectively.

To evaluate the dataset, we compare it to the two networks with different scales, as shown in Fig. 5(a–d). For the smaller and larger networks, the R of our dataset is 0.86 and 0.85, respectively, higher than the original product (0.85 and 0.80, respectively), also with more available values. For non-ECV coverage period, the R is 0.22 and 0.33 for the small and large grid, respectively, showing significant correlation (P = 0.05). This indicates that the dataset has the comparable accuracy with the original dataset, but much better spatio-temporal continuity.

### The trend of soil moisture

**Fig. 6:**
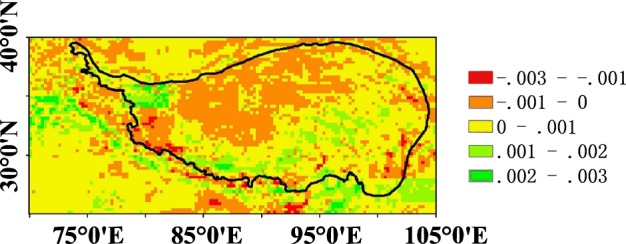
Spatial patterns of the estimated trend for soil moisture over the TP, 2002–2015 based on the proposed soil moisture dataset unit: cm^3^ cm^-3^ per year.

The trends of soil moisture over TP during 2002–2015 are analysed with the least square method^17^ and shown in Fig. 6. It can be seen that most parts of the TP (68.6%) has an increase trend (Fig. 6), indicating wet trend. This is consistent with the results based on the long-time series of *in-situ* measurements^18^. The areas with a soil moisture decreasing trend are mainly distributed in north-inner of the Plateau, nearby the Taklimakan Desert, the biggest desert of China. It also can be seen that the extreme increasing and decreasing trends seen around the southern and south-eastern boundary areas. Soil moisture over the TP is undergoing significant change along with global change.

## Data Availability

Custom code for handling the dataset and all input data are available at Data Citation^15^. This Matlab code enables users to easily reconstructed the soil moisture product. Core of the code is the application of GRNN model. The code requires Matlab version 2016b or higher.
